# In Vivo Analysis of the Role of O-Glycosylations of Von Willebrand Factor

**DOI:** 10.1371/journal.pone.0037508

**Published:** 2012-05-17

**Authors:** Idinath Badirou, Mohamad Kurdi, Paulette Legendre, Julie Rayes, Marijke Bryckaert, Caterina Casari, Peter J. Lenting, Olivier D. Christophe, Cecile V. Denis

**Affiliations:** 1 Institut National de la Santé et de la Recherche Médicale U770, Le Kremlin-Bicêtre, France; 2 UMR_S 770, Univ Paris Sud, Le Kremlin-Bicêtre, France; National Cerebral and Cardiovascular Center, Japan

## Abstract

The objective of this project was to study the function of O-glycosylations in von Willebrand factor (VWF) life cycle. In total, 14 different murine *Vwf* cDNAs mutated on one or several O-glycosylations sites were generated: 9 individual mutants, 2 doublets, 2 clusters and 1 mutant with all 9 murine glycosylation sites mutated (Del-O-Gly). We expressed each mutated cDNA in VWF deficient-mice by hydrodynamic injection. An immunosorbent assay with Peanut Agglutinin (PNA) was used to verify the O-glycosylation status. Wild-type (WT) VWF expressed by hepatocytes after hydrodynamic injection was able to bind PNA with slightly higher affinity than endothelial-derived VWF. In contrast, the Del-O-Gly VWF mutant did not bind PNA, demonstrating removal of O-linked glycans. All mutants displayed a normal multimeric pattern. Two mutants, Del-O-Gly and T1255A/T1256A, led to expression levels 50% lower than those induced by WT VWF and their half-life in vivo was significantly reduced. When testing the capacity of each mutant to correct the bleeding time of VWF-deficient mice, we found that S1486A, T1255A, T1256A and the doublet T1255A/T1256A were unable to do so. In conclusion we have shown that O-glycosylations are dispensable for normal VWF multimerization and biosynthesis. It also appears that some O-glycosylation sites, particularly the T1255 and T1256 residues, are involved in the maintenance of VWF plasma levels and are essential for normal haemostasis. As for the S1486 residue, it seems to be important for platelet binding as demonstrated in vitro using perfusion experiments.

## Introduction

Von Willebrand Factor (VWF) is a large multimeric plasma glycoprotein essential for normal haemostasis. Its main role is to mediate platelet adhesion to exposed subendothelial tissues at sites of vascular injury [1], and its accessory role is to act as a carrier molecule for procoagulant factor VIII, thereby protecting it from premature clearance [2].

During its synthesis which takes place exclusively in megakaryocytes and endothelial cells, VWF undergoes extensive post-translational modifications such as dimerisation, removal of the propeptide, multimerization and addition of polysaccharidic chains [3]. There are 12 N-linked and 10 O-linked glycosylation sites per mature monomer [4]. In VWF, these carbohydrates account for approximately 20% of the molecular weight of the protein.

Polysaccharidic chains have been shown to contribute to a number of cellular processes such as protein folding, stability and secretion but they can also influence the biological activity and survival of the molecule [5], [6], [7].

For VWF, the influence of glycans in its life cycle has long been known. Indeed it has been reported in 1986 that inhibition of the attachment of the precursor N-glycan structure to the protein backbone results in a complete inhibition of initial dimerization of VWF protomers and subsequent targeting to the Golgi [8]. A recent article specifically identified four glycosylation sites (one in the propeptide and three in the mature subunit) involved in this process [9]. N-glycans are also known to carry ABO blood group determinants, thus directly influencing VWF levels which are 25% lower in people with blood group O compared to non-O, due to an accelerated clearance [10]. Another recently described role for N-glycosylation in VWF relates to its potential to modulate VWF interaction with ADAMTS13 and subsequent proteolysis [11].

Although there is ample evidence for the influence of N-glycosylation in VWF biology and function, it is much less clear for O-glycosylation. O-linked glycosylation is characterized by the addition of N-acetyl-galactosamine (GalNAc) to a serine or a threonine residue followed by other carbohydrates such as galactose and sialic acid. In human VWF, the predominant O-linked glycan consists of the sialylated Gal-(β1–3)-GalNAc (also known as T-antigen) which represents 70–90% of all O-linked glycan structures. A recent study on the O-glycome of VWF revealed that a small portion of the O-linked carbohydrates is characterized by the presence of ABO blood group structures [12]. A few years ago, we have reported that O-linked glycans could contribute to the regulation of VWF plasma levels since an inverse correlation was found between the amount of sialylated T-antigen and plasma levels of VWF [13]. An important aspect of O-linked glycosylations is their clustering around the A1 domain, which carries the platelet binding site. Indeed, eight out of the ten O-glycosylation sites of VWF are surrounding this domain. This observation prompted some investigators to study their influence on VWF-platelet interaction. In 1992, by producing recombinant VWF in Chinese Hamster Ovary (CHO) cells allowing selective suppression of O-linked glycosylations, Carew et al suggested that these structures were important for optimal binding of VWF to platelet glycoprotein (GP) Ib in the presence of ristocetin [14]. In 2005, another study using a number of isolated recombinant A1 domains carrying mutations at O-glycosylation sites, reported either enhanced or decreased platelet interaction according to the mutation examined [15].

In view of these few and inconclusive studies, we undertook the characterization of the functional role of VWF O-glycosylation sites in an *in vivo* model. Mutations were introduced to abolish O-glycosylations at specific sites in murine *Vwf* cDNA and the different mutants were expressed in VWF-deficient mice by hydrodynamic injection.

## Methods

### Ethics statement

The animal facility as well as the protocols used throughout the study were validated by the Veterinary services under the French Minister of Agriculture authority. Our official agreement number is B94-043-13.

### Mice

Approximately eight week-old VWF-deficient (VWF^−/−^) mice on a C57BL/6 background bred in the INSERM U770 animal facility, were used throughout this study. Housing and experiments were done as recommended by French regulations and the experimental guidelines of the European Community.

### Hydrodynamic injection of plasmid DNA

Plasmid DNA (100 µg or quantities indicated in individual experiments) diluted in a volume of saline (0.9%) equivalent to 10% of the bodyweight were injected into the mouse tail vein within 5 seconds. A 2 ml syringe with a 26^½^ gauge needle was used.

### Selection and construction of O-glycosylation mVWF mutants

O-linked glycosylation sites on human VWF were identified in 1986 by Titani et al [4]. Experimental evidence was based on the lack of identifiable phenylthiohydantoins after Edman degradation at known threonine/serine sites, associated with the presence of proline residues in the vicinity. No such studies have been performed with murine VWF (mVWF) and therefore the presence of O-linked glycosylations at conserved sites can be questioned. Of the 10 threonine/serine residues reported to carry O-glycosylation on human VWF cDNA, 9 are conserved in m*Vwf* cDNA (Table 1). We first used an internet-server-based neuronal network for prediction of mucin type GalNac O-glycosylation sites in mammalian proteins (http://www.cbs.dtu.dk/services/NetOGlyc-3.0/). However, using human VWF primary sequence as a test sequence, we did not get satisfying results since only 5 out of the 10 sites identified by Titani et al were predicted to be O-glycosylated and one additional site, not identified by Titani, was considered a putative site for O-glycosylation using the online tool. We therefore decided to make the assumption that when the threonine/serine residue was conserved between human and mouse, we would consider that this residue was O-glycosylated. This assumption is based on the fact that there is normally very little evolutionary pressure to conserve site-specific mucin-type glycosylated serines and threonines [16]. So the observation that residues predicted to be O-glycosylated in the human VWF sequence are very well conserved in sequences of different species (see Table 1) would argue in favor of a specific role of these residues and therefore would justify their conserved O-glycosylation status.

**Table 1:** Conservation of residues known to carry O-glycosylations on human VWF in a number of animal species.

Human	T1248	T1255	T1256	S1263	T1468	T1477	S1486	T1487	T1679	T2298
Mouse	-	-	-	**P1263**	-	-	-	*S1487*	-	-
Rat	-	-	-	**P1263**	-	-	-	*S1487*	-	-
Cow	-	-	-	**P1263**	-	-	-	**L1487**	-	*S2298*
Rabbit	-	-	**I1256**	**E1263**	-	-	-	**P1487**	-	-
Cat	-	-	-	**P1263**	-	-	-	*S1487*	-	-
Dog	-	-	-	**P1263**	-	-	-	*S1487*	-	-
Horse	-	-	**I1256**	**P1263**	-	-	-	**P1487**	-	-
Chimpanzee	-	-	-	-	-	-	-	-	-	-
Boar	-	-	-	P1263	-	-	-	-	-	-
Pig	-	-	-	P1263	-	-	-	-	-	-

In bold are residues that are not conserved and in italics are residues that are modified but could still carry O-glycosylations (change of a threonine to serine). Residues are conserved when nothing is indicated.

Full-length murine *Vwf* (m*Vwf*) wild-type (WT) cDNA inserted into a pLIVE expression vector (Mirus Bio, Madison, WI) was used as template for the introduction of mutations [17]. 14 mutants were generated: 9 single mutants, 2 double mutants (T1255A/T1256A and S1486A/S1487A), 2 clusters surrounding the A1 domain (T1248A/T1255A/T1256A and T1468A/T1477A/S1486A/S1487A) and 1 mutant deleted of all 9 O-glycosylation sites (Del-O-Gly). Mutant Del-O-Gly was constructed in three subsequent steps. First, a fragment containing mutations T1468A/T1477A/S1486A/S1487A (cluster 2) was ligated into the cDNA containing the mutations T1248A/T1255A/T1256A (cluster 1). This cluster 1/cluster 2 construct was then used as a template to incorporate the T1679A mutation. Finally, the cluster 1/cluster 2/T1679A construct was used as a template to introduce the T2298A mutation. WT, T1255A/T1256A and Del-O-Gly m*Vwf* cDNAs were also subcloned in the pNUT vector for transfection of COS-7 cells. pNUT is an expression plasmid described previously [18].

Two different techniques of site-directed mutagenesis were used for generation of the mutants, overlap extension using the polymerase chain reaction or QuikChange II XL Site-Directed Mutagenesis Kit (Stratagene, Agilent Technologies France, Massy, France). All Serine or Threonine residues were mutated in Alanine and the primers used for the mutations are summarized in table 2.

To check for the absence of undesired mutations all 14 mutants were entirely sequenced using either the ABI PRISM Dye Terminator Cycle Sequencing Reaction Kit v3.1 (Applied Biosystems, Applera, Courtaboeuf, France) on an ABI PRISM 310 DNA sequencer according to the manufacturer's specifications or using the Genomics services company COGENICS (Takeley, Essex, United Kingdom). Plasmid DNA was amplified in *Escherichia coli* DH5α cells and purified by a Nucleobond endotoxin-free plasmid DNA PC 2000 kit (Macherey-Nagel, Hoerdt, France).

### Cell culture and transfection

COS-7 were grown in Dulbecco's modified eagle medium (DMEM) with 4.5 g/l glucose containing 2 mM L-glutamine, 100 U/ml penicillin, 100 µg/ml streptomycin (all from Invitrogen, Cergy Pontoise, France) and 10% fetal calf serum (FCS) (Biowest, Nuaille France). COS-7 were transiently transfected by electroporation (255 Volts, 1500 µF, ∞ Ohm) using 10 µg of a pNUT expression vector containing m*Vwf* cDNA either WT or mutated (T1255A/T1256A or Del-O-Gly). Transfected COS-7 cells were incubated for 24 hours in MCDB medium (Sigma, Saint Quentin Fallavier, France) with 10% FCS. Then, the medium was changed to serum-free MCDB medium and harvested after 72 hours. Protease inhibitors (1 mM benzamidine, 0.5 mM 4-2-aminoethyl-bezensulfonylfluoride-HCl and 1 mM N-ethylmaleimide) were added to the culture media and the mix was centrifuged for 20 minutes at 4,000 g to remove cells debris. Transfected COS-7 cells were washed twice in PBS and lysed after 5 minutes incubation at room temperature with 1.5 ml lysis buffer (10 mM Tris-HCL pH 8.0, 150 mM NaCl, 5 mM EDTA, 1% Nonidet P-40) containing protease inhibitors. Culture medium and lysate of COS-7 transfected by each m*Vwf* cDNA were used for subsequent analysis.

### Bleeding time assay

Four to five days after hydrodynamic injection, mice were anesthetized with tribromoethanol 2.5% (0.15 ml per 10 g of body weight) and 3 mm of the distal tail were cut using a scapel. The amputated tail was immersed immediately in a 50 ml tube containing physiologic saline at 37°C. Bleeding time was measured from the moment of transection until first arrest of bleeding. Observation was stopped at 600 seconds when bleeding did not cease. VWF expression levels were determined in all mice, and only those expressing 300–1000% were included in this analysis.

### Blood collection and determination of murine VWF antigen levels by enzyme-linked immunosorbent assay

Mice were anesthetized with tribromoethanol 2.5%, and blood was collected from the retroorbital venous plexus into plastic tubes containing trisodium citrate 1.138M (9∶1, v∶v). To obtain platelet-poor plasma, blood samples were centrifuged at 1500 g for 20 minutes at 22°C. Plasma VWF concentration was measured according to a previously described immunosorbent assay using a polyclonal antibody anti-human VWF (Dako France SAS, Trappes, France) and a horseradish peroxidase-conjugated polyclonal antibody anti-human VWF (Dako) [19]. These polyclonal anti-human VWF antibodies cross-react with murine VWF and can be used for the analysis of murine VWF plasma levels [20]. Normal pooled plasma from 20 C57BL/6 WT mice was used as reference and set at 100%. Results were expressed as a percentage of normal murine VWF level.

### VWF multimer structure analysis

The multimeric structure of VWF was analysed by 0.1% sodium dodecyl sulfate (SDS) and 2% agarose (GE Healthcare, Velizy, France) gel electrophoresis [21]. Multimers were visualized using an alkaline phosphatase-conjugated anti-human VWF polyclonal antibody.

### Clearance of VWF

Four days after hydrodynamic injection of VWF-deficient mice with selected O-glycosylation mutants, mice were injected intravenously within the tail vein with 500 µg of biotin-N-hydroxysuccinimide ester (NHS, Calbiochem, Merck Chemicals Ltd, Nottingham, United Kingdom) dissolved in saline buffer [17]. The biotinylation reaction was allowed to complete for 10 min and blood was collected (100 µl), corresponding to time = 0. Additional collections were performed at 0.5, 1, 2, 6 and 24 hours after t = 0. Three mice were used for each time point. Residual biotinylated VWF was measured by an immunosorbent assay, using polyclonal anti-human VWF antibodies (Dako) and horseradish peroxidase-labeled streptavidin (R&D Systems Europe, Lille, France) and was expressed as the percentage of biotinylated VWF levels at t = 0. For each mouse, samples taken at t = 0 were set at 100%, which served as a reference for later time points. Data were fitted with the use of GraphPad Prism (Version 5 for Mac OSX; GraphPad Software).

**Table 2:** List of primers used for generation of O-linked glycosylation mutants.

PRIMERS NAMES	NUCLEOTIDE SEQUENCES
T1248AVWFmuS	5′GCCTGGTCGCCCCCCCC**GCA**GATGCCCCAGTCAGC 3′
T1248AVWFmuAS	5′GCTGACTGGGGCATC**TGC**GGGGGGGGCGACCAGGC 3′
T1255AVWFmuS	5′GTCAGCTCT**GCC**ACCCCATATGTT 3′
T1255AVWFmuAS	5′AACATATGGGGT**GGC**AGAGCTGAC 3′
T1256AVWFmuS	5′GTCAGCTCT**GCC**ACCCCATATGTT 3′
T1256AVWFmuAS	5′AACATATGG**GGC**GGTAGAGCTGAC 3′
T1468AVWFmuS	5′GCCCCAGCCCCA**GCT**CAGCCTCCACAG 3′
T1468AVWFmuAS	5′CTGTGGAGGCTG**AGC**TGGGGCTGGGGC 3′
T1477AVWFmuS	5′GTAGCCCATGTC**GCC**GTGAGTCCAGGG 3′
T1477AVWFmuAS	5′CCCTGGACTCAC**GGC**GACATGGGCTAC 3′
S1486AVWFmuS	5′GCTGGGATC**GCG**TCACCGGGACCA 3′
S1486AVWFmuAS	5′TGGTCCCGGTGA**CGC**GATCCCAGC 3′
S1487AVWFmuS	5′GCTGGGATCTCG**GCA**CCGGGACCA 3′
S1487AVWFmuAS	5′TGGTCCCGG**TGC**CGAGATCCCAGC 3′
T1679AVWFmuS	5′CAACTGCCC**GCC**CTCCCCCCTCTC 3′
T1679VWFmuAS	5′GAGAGGGGGGAG**GGC**GGGCAGTTG 3′
T2298AVWFmuS	5′CCGTGTCCC**GCA**GCCCGAGCTCCC 3′
T2298AVWFmuAS	5′GGGAGCTCGGGC**TGC**GGGACACGG 3′
T1255–T1256AVWFmuS	5′GTCAGCTCT**GCCGCC**CCATATGTT 3′
T1255–T1256VWFmuAS	5′AACATATGG**GGCGGC**AGAGCTGAC 3′
S1486– S1487VWFmuS	5′GCTGGGATC**GCGGCA**CCGGGACCACAG 3′
S1486–S1487VWFmuAS	5′CTGTGGTCCCGG**TGCCGC**GATCCCAGC 3′

Mutated nucleotides are written in bold.

### Immunosorbent assay for VWF-linked T antigen to detect O-linked sugars on VWF

96 wells plates (Greiner Bio-One SAS, Courtaboeuf, France) were coated with polyclonal anti-VWF antibodies 3 µg/ml (Dako) for 2 hours at 37°C and post-coated 1 hour at 37°C with PBS/BSA 3%. Wells were washed 3 times with PBS/0.1%Tween-20 (PBS/T) and incubated 3 hours at 37°C with dilutions of plasma samples from mice injected hydrodynamically with selected O-linked glycosylation mutants. A preliminary step to measure VWF expression level had been performed in order to adjust antigen concentration to 100% in all plasma samples. After washing 3 times with PBS/T, wells were incubated with 5 mU/ml neuraminidase (Roche Diagnostics, Meylan, France) in PBS/1 mM CaCl_2_ overnight at 37°C. After 3 washes with PBS/T, non-specific binding sites were then blocked with the avidin/biotin blocking kit (Vector Laboratories, Burlingame, CA) according to the manufacturer's instructions. After washing 3 times with PBS/0.1%Tween-20, biotinylated peanut agglutinin (btPNA) (Vector Laboratories) in PBS/0.1 mM CaCl_2_ was added for 2 hours at 37°C. Finally, wells were incubated with HRP conjugated streptavidin. Bound btPNA was detected by measuring HRP activity using OPD as substrate [13]. A similar experimental approach in which the de-sialylation step was omitted was also used to test binding of two other biotinylated lectins, Erythrina Cristagalli (ECL, which recognizes terminal Galactose) and Wheat Germ Agglutinin (WGA, which recognizes terminal N-acetylglucosamine and sialic acids). Both lectins were obtained from Vector Laboratories.

### Thrombus formation under flow

Blood perfusion experiments were performed in a parallel plate perfusion chamber essentially as described previously [22]. PPACK (80 µM) anticoagulated blood from VWF-expressing mice was incubated with rhodamine 6G (10 µg/ml) for 5 min at 37°C and then perfused on glass coverslips pre-coated overnight at 4°C with fibrillar equine type 1 collagen (100 µg/ml) at a shear rate of 2500 s^−1^ with a syringe pump (Fisher Scientific, Ottawa, Canada) for 1 min. Unbound material was removed by a subsequent perfusion with Hepes-Tyrode buffer for 30 sec. Thrombus formation was recorded with an inverted epifluorescence microscope (Nikon Eclipse TE2000-U) coupled to the Metamorph-7.0rl software (Universal Imaging Corporation) and was quantified by the assessment of the mean percentage of the total area covered by platelets using ImageJ-1.44 software (http://rsbweb.nih.gov/ij/index.html).

## Results

### Detection of O-linked sugars on VWF expressed in mice after hydrodynamic injection

**Figure 1:**
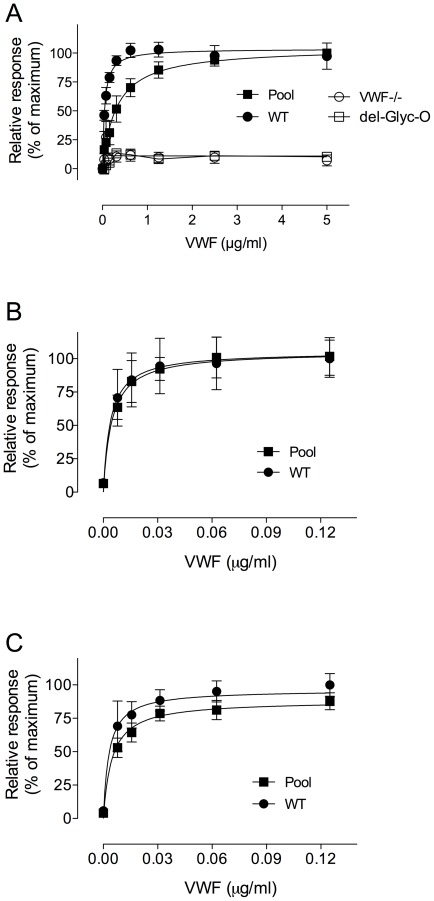
Detection of T antigen on VWF in murine plasma. Microtiter wells coated with polyclonal anti-VWF antibodies (3 µg/ml) were incubated with serial dilutions of murine plasma previously adjusted for VWF concentration. After catching VWF, wells were incubated with neuraminidase (5 mU/ml) in PBS/1 mM CaCl_2_ overnight at 37°C (neuraminidase incubation was omitted for data presented in panels B and C). Subsequently, wells were blocked with the avidin/biotin blocking kit and incubated for 2 hours at 37°C with btPNA (panel A), btECL (panel B) or btWGA (panel C). After washing, HRP-conjugated streptavidin was added to the wells and bound lectin was detected by measuring HRP activity using OPD as a substrate. *Panel A:* btPNA binding to pooled plasma of C57Bl6J mice (Pool), plasma of WT-VWF expressing mice (WT), plasma of Del-O-Gly expressing mice (Del-O-Gly) and plasma of VWF-deficient mice (VWF−/−); *Panel B:* btECL binding to Pool and WT; *Panel C:* btWGA binding to Pool and WT. Plotted is the relative response (% of maximum binding) versus concentration of VWF in diluted samples. The relative response is defined as binding being relative to binding of the lectins to WT VWF (5 µg/ml for btPNA and 0.125 µg/ml for btECL and btWGA), which was arbitrarily set at 100%. Data points represent the mean±SD of 3–6 measurements.

In VWF, the predominant O-linked glycan consists of the sialylated T-antigen [23]. A few years ago, we developed a specific assay using btPNA, a lectin that specifically recognizes the non-sialylated T-antigen and we were able to measure the amount of O-linked sugars on VWF in various plasma samples from patients [13]. In the present study we first adapted the test for murine plasma samples. Then, considering that VWF produced after hydrodynamic injection is synthesized by hepatocytes and not by endothelial cells or megakaryocytes, we wanted to check whether similar glycosylation structures were present on the molecule. We first used the btPNA assay to compare normal pooled plasma from WT mice to plasma samples from VWF-deficient mice injected hydrodynamically with WT-m*Vwf* cDNA. As shown in Fig. 1A, btPNA bound more avidly to WT-VWF from plasma of mice after hydrodynamic injection (Half-maximum binding 3.2±0.4 µg/ml and 0.5±0.1 µg/ml for normal pooled plasma and WT-VWF, respectively; *p*<0.0001), potentially suggesting a better access of the lectin to the O-linked glycans in this setting. However, the similar Bmax value indicated that an equivalent number of binding sites were present in both samples. As a negative control, plasma from a VWF-deficient mouse was tested and no binding to btPNA could be observed. In addition, no btPNA binding was observed to mice expressing the Del-O-Gly variant in which all 9 potential O-linked glycosylation sites were mutated. This suggests, that besides the 9 O-linked sites, no additional glycosylation sites are present that contain the Gal-(β1–3)-GalNAc structure. In a second series of controls, we also examined the binding of lectins ECL and WGA, which recognize terminal galactose and sialic acids, respectively. Both lectins bound efficiently to VWF from normal C57Bl6 mice (Figs. 1B and 1C, respectively). Efficient binding was also observed for WT-VWF from plasma of mice after hydrodynamic injection, with both Bmax and half-maximal binding being similar to those of normal VWF. Apparently, a similar extent of terminal galactose and sialic acids are exposed on endothelial- and hepatic-derived VWF.

### Multimeric analysis of O-glycosylation mutants

**Figure 2:**
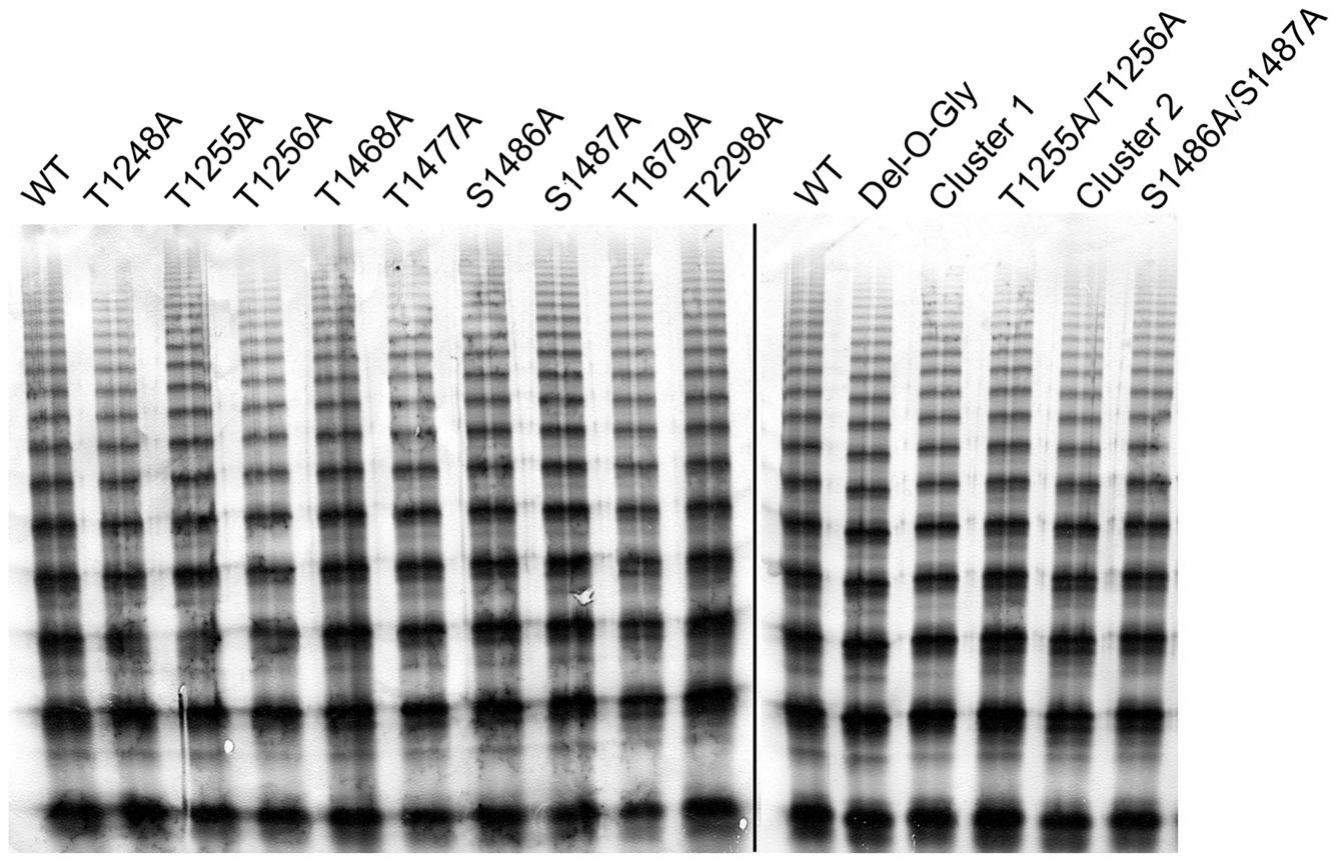
Multimeric analysis of O-glycosylation mutants. VWF-deficient mice were injected with 100 µg of pLIVE-m*Vwf*, WT or the various O-glycosylation mutants. Plasma was collected 96 hours later and analysis of plasma samples was performed by SDS/2% agarose gel electrophoresis.

In order to study the potential role of O-linked glycosylation on VWF multimerization, we loaded plasma samples from VWF-deficient mice injected with WT or mutated m*Vwf* cDNA on 2% agarose gel. Fig. 2 shows the profile obtained with all 14 mutants as compared to WT cDNA. Faster migration is visible for the Del-O-Gly and also for the Cluster 2 mutant, demonstrating that we have indeed removed glycosylations normally contributing to the molecular mass of VWF. All mutants, including the Del-O-Gly construct led to the production of multimers covering the entire range of molecular weight. This was confirmed via densitometric analysis of 2 (for 6 mutants) or 3 (for 8 mutants) independent multimer patterns. For WT, we analyzed 7 independent multimer patterns. Multimer distribution for WT was as follows: 1–5 multimers: 71±4% of total multimers, 6–10 multimers: 22±4%, 11–15 multimers: 6±1%, >15 multimers: 1.0±0.4%. A similar distribution was found for all mutants (1-way ANOVA: *p*>0.05 for each of the multimer ranges).

### VWF expression levels after hydrodynamic injection of O-glycosylation mutants in VWF-deficient mice

**Figure 3:**
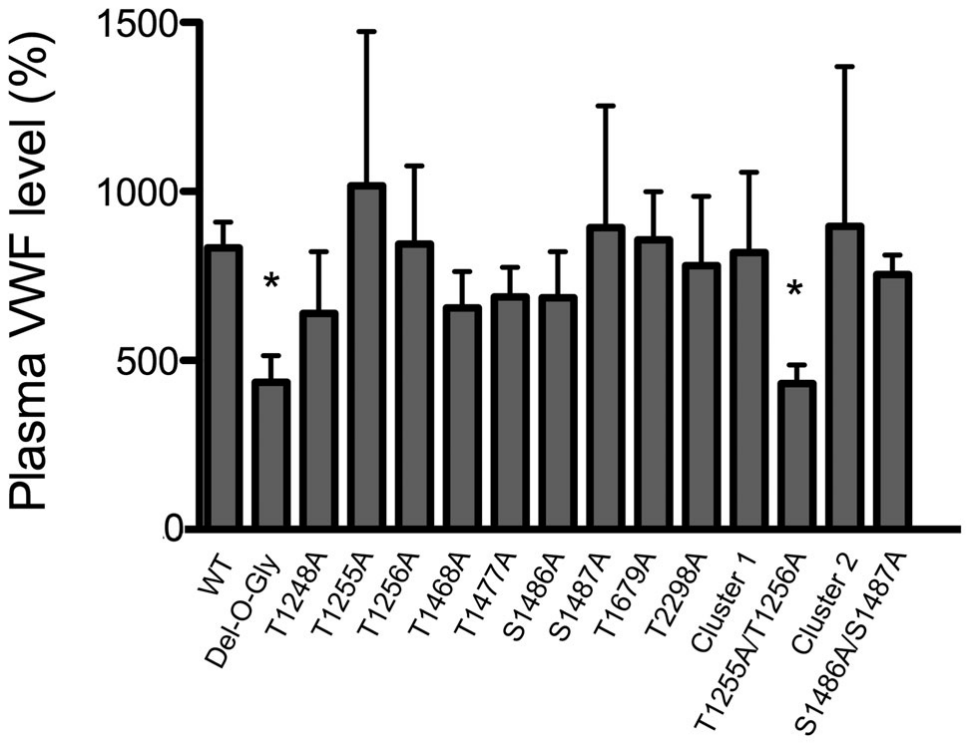
VWF∶Ag expression levels following hydrodynamic gene delivery. VWF-deficient mice were injected with 100 µg of pLIVE-m*Vwf*, WT or the various O-glycosylation mutants. Plasma was collected 96 hours later and VWF∶Ag levels were measured by ELISA. Data are represented as mean plus or minus SEM. Normal pooled plasma from 20 C57BL/6 mice was used as a reference and set at 100%. Results are expressed as a percentage of a normal murine level. n = 22 for WT cDNA and 4–5 for the various mutants. * p<0.05 calculated with unpaired t-test when comparing the mutant with WT cDNA.

To investigate the consequences on VWF expression levels of removing one or more O-linked glycosylation sites, we injected all 14 mutants in VWF-deficient mice by hydrodynamic injection (100 µg of cDNA) and we measured VWF antigen 4 to 5 days after the injection. We previously showed that the technique using the pLIVE vector allows a stable expression for at least two weeks [24]. All mutants were compared to WT m*Vwf* cDNA, which is expressed at a mean level of 833±76%. As shown in Fig. 3, 12 out of 14 mutants were expressed at levels very close to the WT, not showing any statistical differences. In contrast, two mutants led repeatedly to lower expression levels: the T1255A/T1256A doublet (432±55%, p<0.05 as compared to WT cDNA) and the Del-O-Gly mutant with complete removal of all 9 O-linked glycosylation sites (435±80%, p<0.05 as compared to WT cDNA).

### Cellular transfection of mutants with low expression levels

**Figure 4:**
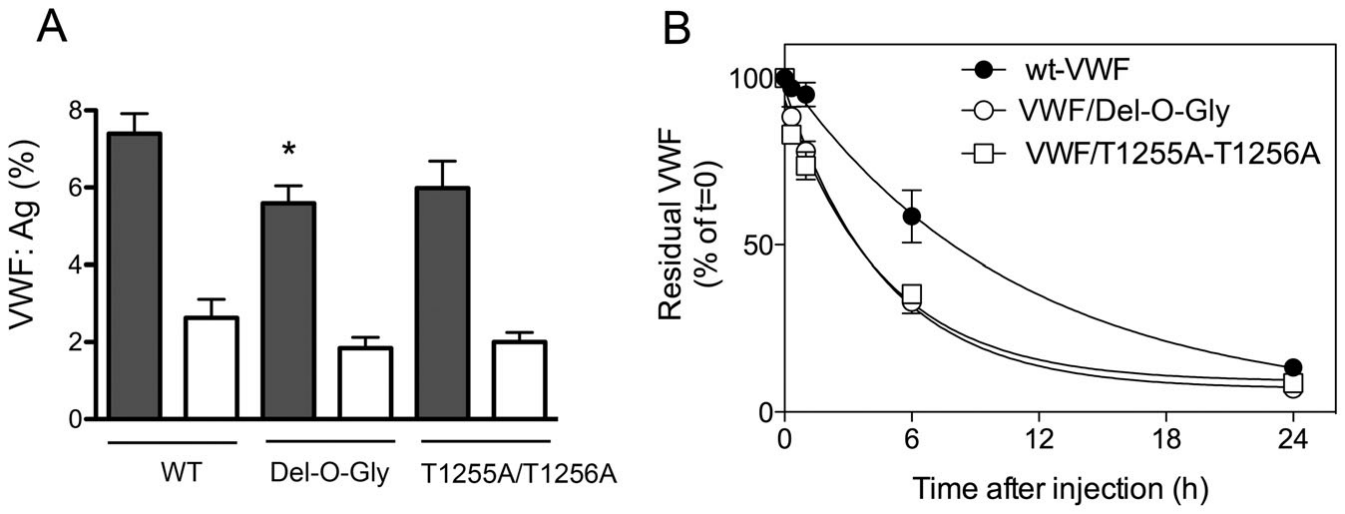
Cellular expression and in vivo clearance of O-glycosylation mutants. *Panel A:* pNUT vectors containing WT-m*Vwf* cDNA, T1255A/T1256A or the Del-O-Gly mutant were transfected in COS-7 cells by electroporation. 96 hours later, cell supernatants were collected and cells were lysed. VWF antigen levels were measured by ELISA in the supernatants (grey bars) and lysates (white bars) for each mutant and the WT mVWF. n = 7–8 individual transfections, * p = 0.022 using unpaired t-test when comparing to WT. *Panel B:* After injection with NHS-biotin, residual biotinylated VWF was determined at indicated time points. Data present the percentage of residual biotinylated VWF measured at t = 0, which was set at 100% for each mouse. Curves indicate the best fit for an exponential decay.

To investigate if lower synthesis could contribute to the low expression levels obtained with the T1255A/T1256A and Del-O-Gly mutants, we performed transient transfections in COS-7 cells with these two mutants and WT m*Vwf*. After transfection by electroporation, VWF antigen levels were measured in both the cell lysate and the supernatant (Fig. 4A). We observed lower VWF expression in the cell supernatant of the Del-O-Gly mutant compared to WT (p = 0.022). However, no intracellular retention could be detected. For the T1255A/T1256A mutant, no difference in cell supernatant or cell lysate was measured compared to WT (p = 0.12 for the supernatant).

### Clearance of O-linked glycosylation mutants in mice

We also investigated whether the low expression levels in the T1255A/T1256A or the Del-O-Gly mutants could result from increased clearance. m*Vwf* cDNA (WT or mutant) were injected via hydrodynamic injection in VWF-deficient mice. Four days later, mice were injected with NHS-biotin to label circulating VWF. Subsequently, samples were taken at indicated time points and residual biotinylated VWF in plasma was determined. Elimination of WT-mVWF from the circulation followed a single exponential decay with a calculated half-life of 6.1±1 hour (95% confidence interval [CI], 4.2–10.9 hours; Fig. 4B). A single-exponential decay was also observed for both mutants. However, half-life was significantly shorter for both mutants compared to WT-mVWF (p = 0.0011): 3.0±0.3 hours (95% CI, 2.4–4.2 hours) for the T1255A/T1256A mutant and 2.9±0.2 hours (95% CI, 2.5–3.4 hours) for the Del-O-Gly mutant (Fig. 4B).

### Effect of O-glycosylation mutations on bleeding time

**Figure 5:**
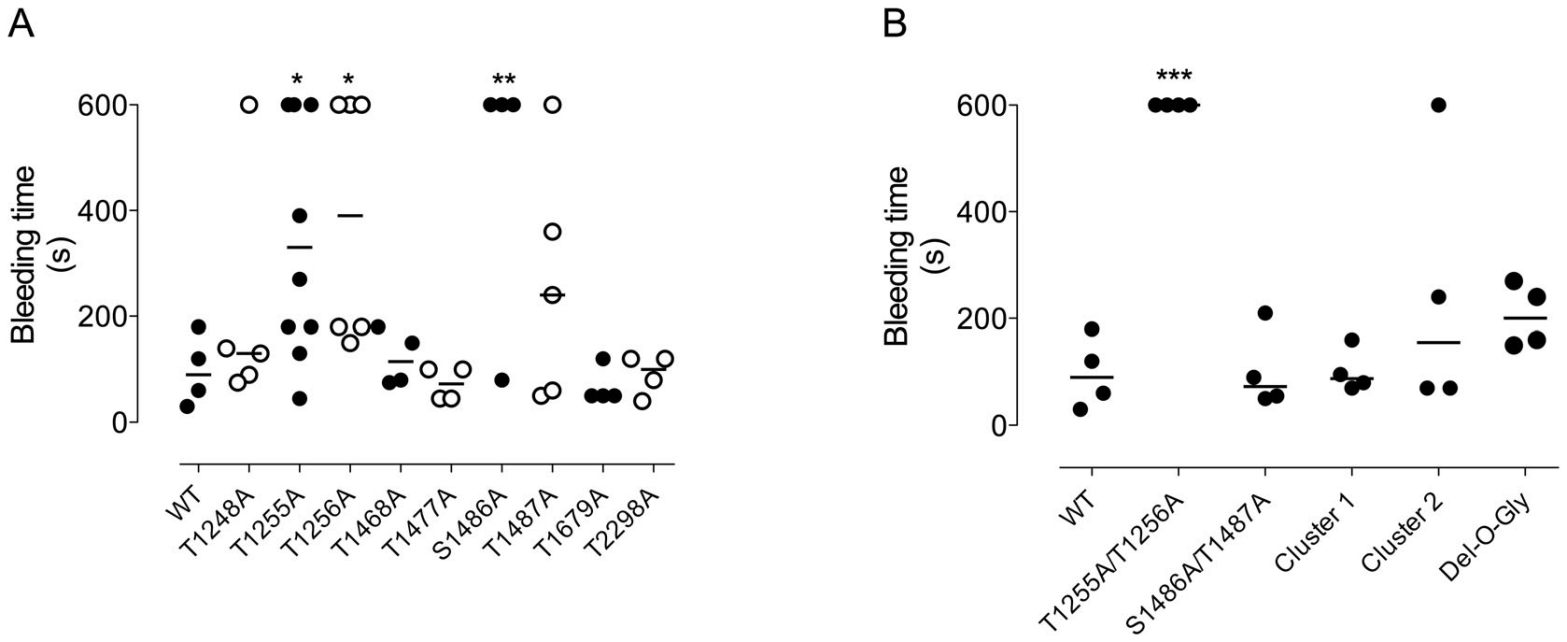
Effect of O-glycosylation mutations on bleeding time. VWF-deficient mice were injected with pLIVE-m*Vwf*, WT or the various O-glycosylation mutants. In order to reach VWF∶Ag expression levels between 300 and 1000%, 100 µg of cDNA was used for WT and most mutants while 150 µg was used for T1255A/T1256A or Del-O-Gly. Four to five days after injection, mice were anesthetized and 3 mm of the tail were cut. The amputated tail was immersed immediately in a 50 ml tube containing physiologic saline at 37°C. Bleeding time was measured from the moment of transection until first arrest of bleeding. Observation was stopped at 600 seconds when bleeding did not cease. *: p<0.05 using unpaired t-test when comparing mutant T1255A or T1256A to WT. **: p = 0.03 using unpaired t-test when comparing mutant S1486A to WT. ***: p<0.0001 using unpaired t-test when comparing mutant T1255A/T1256A to WT.

To evaluate the importance of O-glycosylations on VWF function, we compared the ability of each mutant to correct bleeding time in VWF-deficient mice. In this model, injection of WT-m*Vwf* cDNA leads to bleeding time correction [24], [25]. In contrast, injection of an empty pLIVE-plasmid leaves the bleeding tendency unaffected, with no arrest of bleeding during the 10-min observation period. Among the 9 single mutants, three appeared significantly different from the WT-VWF, with the majority of the mice showing a prolonged bleeding time: T1255A (p = 0.045 *vs* WT), T1256A (p = 0.048 *vs* WT) and S1486A (p = 0.03 *vs* WT) (Fig. 5). When both adjacent residues were mutated (T1255A/T1256A), none of the mice were able to stop bleeding (p<0.001 *vs* WT). This result was independent of the lower expression levels obtained with this particular mutant. Indeed for the bleeding time experiment we only kept mice expressing VWF∶Ag levels between 300 and 1000%, a range where bleeding time correction is systematically obtained with WT-VWF. Interestingly, mutating the residues 1255 and 1256 together with additional O-glycosylations sites, such as the Del-O-Gly or the cluster 1 where residue 1248 is also mutated, abolished the effect observed with the double mutant (Fig. 5). Similarly, mutating S1486A together with its adjacent residue, S1487A or in the context of the cluster 2 or in the Del-O-Gly also abolished the decreased functionality observed with this particular mutant.

### Thrombus formation under flow

**Figure 6:**
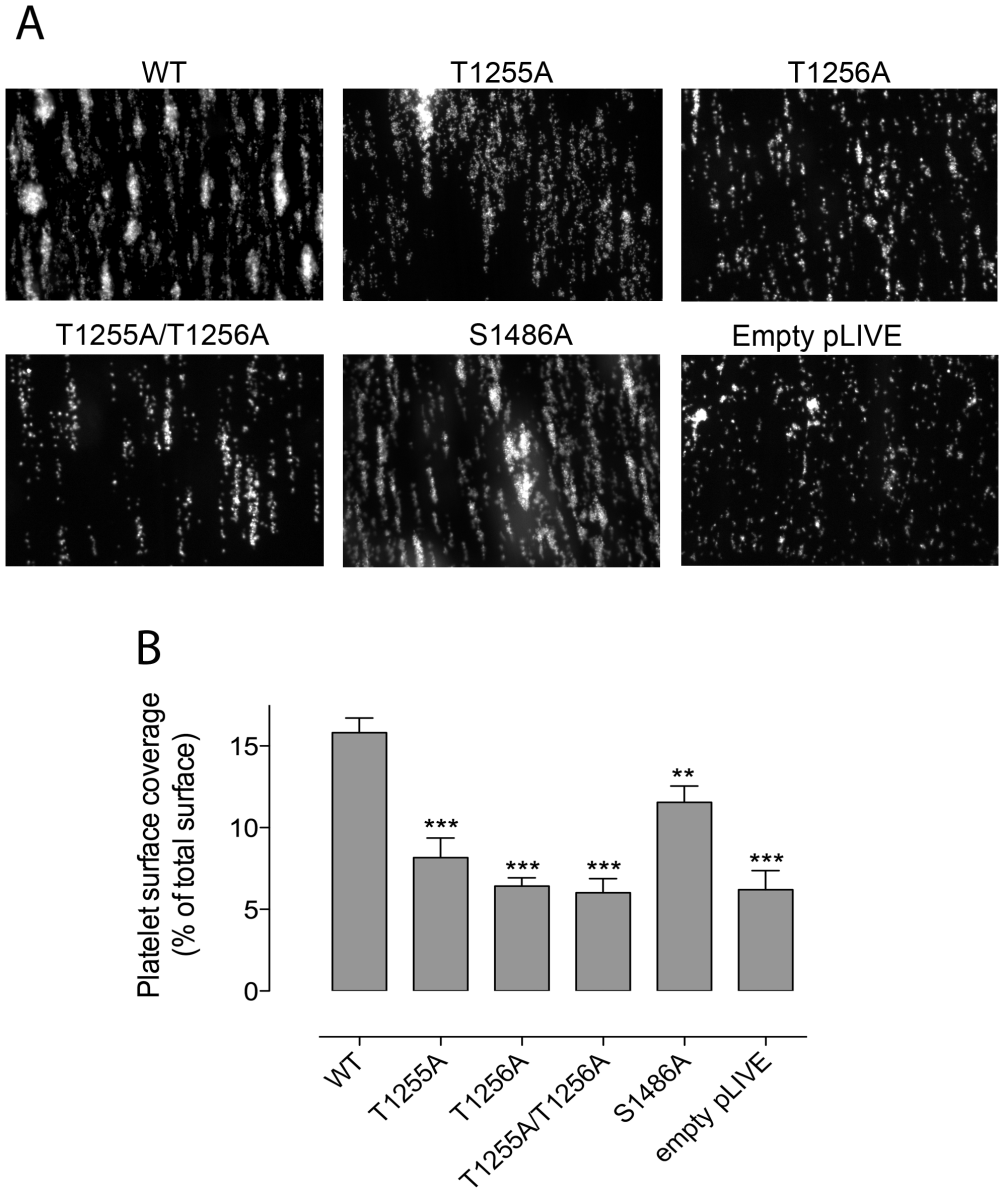
Ex vivo thrombus formation on collagen. *Panel A:* Blood was collected from mice 4 days after hydrodynamic injection with pLIVE encoding WT VWF or one of the following mutants: T1255A, T1256A, T1255A/T1256A, S1486A. As a control, mice were treated with an empty pLIVE plasmid (empty pLIVE). Anticoagulated whole blood was incubated with rhodamine 6G to fluorescently label platelets and perfused over collagen-coated glass coverslips (flow rate 2500 s^−1^) for a period of 1 min. Unbound platelets were removed by subsequent perfusion with Hepes-Tyrode buffer. Thrombus formation was then visualized via image acquisition using Metamorph software. Shown are representative images for WT VWF, each mutant and the negative control (empty pLIVE). *Panel B:* Thrombus formation was quantified using ImageJ software in order to calculate percentages of platelet surface coverage. Data represent the mean±SEM of three independent perfusions, with 3–8 images being analyzed for each perfusion. **: p = 0.006 using unpaired t-test when compared to WT. ***: p<0.0001 using unpaired t-test when compared to WT.

The reduced haemostatic capacity of the mutants associated with a prolonged bleeding time (*i.e.* T1255A, T1256A, T1255A/T1256A and S1486A) was further investigated in a perfusion system in which blood of mice expressing WT or mutant VWF was perfused over a collagen surface at a flow rate of 2500 s^−1^. At such flow rates, adhesion of platelets to collagen is dominated by the action of VWF. Efficient platelet adhesion to collagen was observed when blood of WT-VWF expressing mice was perfused, and platelet-rich thrombi developed homogeneously dispersed over the collagen surface. Platelet surface coverage was calculated to be 15.8±0.9% (mean±SEM; Fig. 6). When blood of VWF^−/−^ mice that were treated with an empty pLIVE plasmid was perfused under identical conditions, a reduction in the number of adhered platelets was observed and few thrombi developed (Fig. 6). Reduced thrombus formation in the complete absence of VWF was corresponding to a significantly reduced platelet surface coverage: 6.2±1.2% (p<0.0001). Interestingly, platelet surface coverage was also reduced for each of the mutants tested: T1255A: 8.2±1.2%; T1256A: 6.4±1.2%, T1255A/T1256A: 6.0±0.9%; p<0.001 for all three mutants; S1486A: 11.6±1.0%, p = 0.006 (Fig. 6). Qualitatively, we observed that all mutants are characterized by the presence of small under-developed thrombi, with few larger thrombi (if any for mutants T1256A and the double mutant). Taken together, the reduced haemostatic capacity of these mutants seems to originate predominantly from a reduced potential to build a thrombus under conditions of arterial shear.

## Discussion

The contribution of O-linked glycans to the properties of glycoproteins is not always well understood and in the particular case of VWF, only very few studies are available in this regard. This lack of information on the role of these carbohydrates in the regulation and function of VWF prompted us to initiate the present study aiming to characterize their contribution in an *in vivo* model. Considering the large number of O-glycosylation sites present on human VWF, we chose the hydrodynamic injection technique to express the different mutants *in vivo* [17], [24], [25]. Indeed, such an approach allowed us to study all individual mutants as well as combinations thereof in VWF-deficient mice. In order to assess functionality, mutagenesis was performed on murine VWF. Out of the 10 O-glycosylation sites present on human VWF, 9 are conserved in the mouse sequence [26] and all 9 were individually replaced by an alanine. Two clusters of 3 and 4 mutations respectively, were also generated as well as two doublets and one mutant with all 9 mutations (Del-O-Gly). VWF-deficient mice were injected with the various m*Vwf* cDNAs and experiments were performed 4–6 days following injection, a time window in which expression levels are stable as we previously showed [24]. Our approach raised two main difficulties. The first one, inherent to any in vivo or in vitro expression system relates to the glycosylation profile of a protein not produced by its original parental cell, which may cause subtle differences in this profile. Unfortunately, with the current technologies available it is not realistic to study the structural and functional consequences of 14 mutations in vivo via endothelial-specific expression of these mutants. So far, studies on the analysis of glycosylation mutants of human VWF have been based on the expression of VWF in heterologous expression systems using CHO- or HEK293-cells [9], [14], [27]. In none of these cells the glycosylation profile of the protein is similar that that found in endothelial-derived VWF. Nevertheless, valuable information was obtained in these studies despite the limitations of the model, indicating that the use of heterologous expression systems is useful in the study of VWF glycosylation. In our system, VWF is produced as a functional multimerized protein. Binding of the lectins ECL (recognizing terminal galactose residues) and WGA (recognizing terminal N-acetylglucosamine and N-acetylneuraminic acid) was similar for both endothelial- and hepatic-derived VWF, indicating that the overall N- and O-linked glycosylation pattern is not dramatically different between both proteins. Moreover, these data indicate that the hepatic-derived VWF is properly sialylated. Indeed, a reduced extent of sialylation would be associated with increased ECL- and decreased WGA-binding. Of course, subtle differences in N-linked glycosylation are not detected using this lectin-based approach and additional studies using mass spectrometrical analysis would be needed in this regard.

As for the O-linked glycans, it is known that recombinant VWF produced in CHO cells as well as plasma-derived VWF contains as the predominant O-linked glycan, a sialylated Gal-(β1–3)-GalNAc structure, representing 70–90% of all O-linked glycan structures [12], [23], [28]. Here we show, using a specific PNA-lectin assay to measure the presence of this Gal-(β1–3)-GalNAc structure, that hepatocyte-derived VWF is not fundamentally different from endothelial-derived VWF in terms of PNA recognition (Fig. 1A). Bmax values indicated very similar number of binding sites for VWF of both origins. In addition, no btPNA binding to Del-O-Gly mutant was detected, suggesting that no additional btPNA recognition sites are present outside the potential O-glycosylation sites that we have mutated. Moreover, we could demonstrate the specificity of the test by the absence of lectin binding to plasma from VWF-deficient mouse. The only difference we could observe between endothelial- and hepatic-derived VWF came from a slightly higher affinity of the lectin for hepatocyte-derived VWF (Fig. 1). The reason for this higher affinity is not completely clear. Since potential differences between hepatic- and endothelial-derived VWF are probably limited to the glycan structures, we anticipate that small differences in surrounding N-linked glycans may contribute to the higher affinity for PNA. It remains difficult to predict how the N-linked glycans elsewhere in the VWF protein are exactly located in comparison to the O-linked glycans in perspective of a three-dimensional structure. It should be noted that the D3 and A2 domains adjacent to the A1 domain (which contains the two O-linked clusters) both contain 2 N-linked glycans. Given that both the D'D3 region and the A2 domain have been reported to cover the A1 domain [29], [30], it is possible that changes in these N-linked glycans modulate the access of btPNA to the O-linked glycans around the A1 domain. This may also be true for the single O-glycan in the B1–3 domain, which contains 4 additional N-linked glycans. Taken together, we feel that our model is not less relevant than other heterologous models [9], [14], [15], [27] to study the effect of glycosylation mutations on VWF structure and function, despite the potential limitations of our in vivo model.

The second issue, specific to our study, related to the use of murine VWF for which no experimental data on the position of O-linked glycosylations, were available. Since online prediction tools did not appear to be reliable for this protein, we assumed that O-glycosylations were present on the same residues in humans and in mice when these residues were conserved. In addition, we assumed that the majority of the glycans consisted of the sialylated T-antigen, as has been found for human VWF [12]. The absence of binding sites for the PNA lectin on the Del-O-Gly variant (Fig. 1) confirms that apart from the 9 selected mutations no other Ser/Thr residues are glycosylated with this Gal-(β1–3)-GalNAc structure, or become so in a compensatory manner. Furthermore, the faster migration of the Del-O-Gly mutant in multimeric gels (Fig. 2) is also compatible with the removal of substantial amounts of glycan structures (molecular weight sialylated T-antigen: 0.7 kDa). Of note, our data do not fully rule the presence of non-T-antigen structures elsewhere in the molecule, but given the data on the number of non-T-antigen structures on human VWF (≤1 *per* subunit) and the loss in molecular mass in Del-O-Gly it seems that few if any of such structures are present in the Del-O-Gly variant. Taken together, in view of all these validation steps we felt confident that our model was pertinent to investigate structure and function of O-linked glycan mutants *in vivo*.

First we checked the contribution of O-linked glycans to VWF multimerization. All 14 mutants, including the Del-O-Gly, were able to multimerize (Fig. 2), suggesting that O-linked glycans are dispensable for this essential step in VWF life-cycle. This result is in accordance with a previous study where recombinant VWF was produced in a cell line with a carbohydrate defect, resulting in the production of fully multimerized VWF specifically lacking O-linked carbohydrates [14]. We next investigated the effect of our mutations on VWF expression levels and found that all mutants were expressed in the mice (Fig. 3). Apparently, O-glycosylations are not a prerequisite for VWF biosynthesis and secretion, which is opposite to the role of some N-glycosylations [8]. Interestingly, two mutants showed repeatedly a 50% decrease in plasma antigen levels: the T1255A/T1256A doublet and the Del-O-Gly mutant. We considered the possibility that reduced antigen levels were associated with a decreased production efficiency. Indeed expression of the Del-O-Gly mutant led to slightly reduced levels of VWF in the extracellular medium compared to WT-VWF using transient transfection in COS-7 cells (Fig. 4). However, no such effect was observed for the T1255A/T1256A doublet. In both cases, the reduced antigen levels following *in vivo* expression was not fully explained by defective biosynthesis. An alternative hypothesis to explain the lower VWF levels in vivo originated from the possibility that these two forms of VWF could be removed quicker from the circulation. Indeed, clearance experiments in mice demonstrated that VWF with two mutations in positions 1255/12566 or with all 9 O-glycosylation sites removed, was eliminated significantly quicker than WT-VWF, accounting for the observed lower plasma levels. This finding is in agreement with our previous study describing a linear relation between PNA binding and propeptide/VWF ratio in humans, suggesting a potential association between O-linked glycosylation and VWF survival [13].

One of the main goals of our study was to investigate the functionality of VWF mutated at specific O-glycosylation sites. The clustering of 8 out 10 of theses sites around the A1 domain in human VWF is particularly intriguing since VWF A1 domain contains the binding site for platelet GPIb. In an attempt to understand the relevance of this structural particularity and therefore the influence of O-linked glycans on platelet binding, previous studies led to conflicting data. A first report described a decreased interaction between VWF lacking O-linked glycans and GPIb when ristocetin was used as a modulator but not when botrocetin was used, suggesting a potential role of O-linked glycans in the interaction with ristocetin rather than directly with GPIb [14]. A second more recent study used a mutagenesis approach to replace specific glycosylation sites with alanine residues [15]. However, this study was done on isolated A1 domain encompassing residues 1236–1476, limiting the evaluation to 4 O-glycosylation sites upstream of the A1 domain and to only 1 site downstream of the A1 domain. The results showed that mutations in the O-linked glycans located in the amino-terminal A1 flanking region increased VWF interaction with GPIb while mutating the 1468 residue in the carboxy-terminal A1 flanking region had the opposite effect [15]. Many questions remained unanswered following these two studies, prompting us to evaluate the capacity of O-glycosylations to regulate VWF function in our *in vivo* model. Our results show that mutation of the T1255/T1256 doublet, of T1255, of T1256 and of S1486 led to non-functional forms of VWF and mice expressing these mutants were unable to correct their bleeding time (Fig. 5). The prolonged bleeding for the T1255/T1256 doublet was not due to decreased levels of VWF antigen in these mice. Like for all other WT and mutants tested, antigen levels were in the range between 300% and 1000%, a range where we observe correction of bleeding with WT systematically. Moreover, expression levels for this double mutant and the Del-O-Gly were near identical (Fig. 3), with the latter being associated with fully corrected bleeding times in all mice. The reduced function of these VWF mutants was confirmed in separate *ex vivo* perfusion studies, in which blood of mice expressing these mutants was perfused over a collagen surface at high shear rates (flow rate 2500 s^-1^). Indeed, all four mutants were associated with reduced platelet surface coverage under these conditions (Fig. 6).

But the most surprising result came from the observation that the cluster mutants and the Del-O-Gly mutant were no different from WT-VWF in their ability to correct bleeding time in VWF-deficient mice. Apparently, the detrimental effect of the absence of a single or doublet glycan structure is rescued by the deletion of additional glycans or even the complete O-glycome. How to explain this observation? At this point we can but speculate on the underlying mechanism, and we would like to share two possible options with the readers. A first possibility is based on the assumption that the O-glycans *per se* do not interact directly with GpIb. This assumption is compatible with the Del-O-Gly being as functional as WT VWF as well as with the fact that *E. coli*-derived A1 domain fragments lacking glycan structures interact efficiently with GPIb [31]. Consequently, the effect of selective deleted O-glycans on GpIb binding is indirect and perhaps related to the charged nature of the glycans. Clusters of sialylated glycans represent a highly negatively-charged region. Selective removal of one or several of these negatively-charged glycans from these clusters may cause a profound charge-dependent re-orientation of the region, which could modulate the interaction with GPIb. Such effects would then be neutralized by the removal of additional glycan structures. A second possibility is based on the assumption that the O-linked glycans do interact with GpIb, but do so in a coordinated manner. In addition, the presence of the glycans prevents GpIb from binding to alternative sites in the VWF protein, which only come available when deleting these glycans. Deletion of one or two glycans from each of the clusters would be insufficient in such case to allow GpIb access to this alternative interactive region. Only the deletion of additional glycans relieves this limitation, thereby restoring VWF-GpIb interactions.

Irrespective of the underlying mechanism, we would like to emphasize that such compensatory mechanisms are not unprecedented. They have for instance been described in perspective of protein mutations. One example hereof have been described by Plaimauer and colleagues, who demonstrated that the detrimental effect of the Arg1336 to Trp mutation on ADAMTS13 biosynthesis is modulated by accompanying polymorphisms in this protein [32]. But also mutations affecting glycosylation sites have been reported in this regard. First, deletion of a single O-linked glycan (T17A) in the activation peptide of coagulation factor X increases the *K*_M_ value for the activation by the tenase complex complex 8-fold [33]. In contrast, such effect is not seen upon deletion of the adjacent O-glycan (T29A). Moreover, deletion of both O-glycans results in normal *K*_M_ values as well, suggesting that deletion of both O-glycans relieves the detrimental effect of deletion of T17 alone. A second example relates to the N-glycans of the HIV-1 gp120 envelope protein [34], which were studied in relation to their role in HIV-1 replication. The replication constant *k* of wt-gp120 was calculated to be 2.961, and mutation of N186Q severely reduced the replication capacity of the virus (*k* = 0.196). However, the deletion of an additional glycan (N136 or N141) restored the replication capacity of the virus (*k* = 2.991 and 2.940, respectively) [34]. Of course, this does not mean that additional deletion of glycans structures always result in compensation. Nevertheless, these data show that it is difficult to predict how single and multiple deletions of glycan sites affect the function of glycosylated proteins.

Taken together, our study has evaluated for the first time the contribution of O-linked glycans to the VWF life cycle. No gross abnormalities with regard to biosynthesis and multimerization are observed when these carbohydrates are missing, suggesting that they are dispensable for these processes. In contrast, a combined mutation of the adjacent T1255/T1256 residues impairs both VWF function and survival. The reduced survival is shared with the variant in which all O-linked glycans are removed, whereas impaired function is shared with the T1255A, T1256 and S1486 single mutants. These data indicate that only a limited number of O-linked glycans are of relevance in the biology of VWF, which is compatible with the notion that so far no mutation affecting O-glycosylation sites has been reported to lead to von Willebrand disease. Finally, studying a VWF variant produced in vitro in an O-linked glycosylation blocking environment may not be predictive of the individual effect of each O-glycosylation site since we show here that specific sites may have more impact when affected alone that in combination with other sites.
